# Participation of teenagers with vision or motor impairments in leisure activities: a qualitative study

**DOI:** 10.3389/fresc.2024.1444901

**Published:** 2024-11-25

**Authors:** Carlijn Veldhorst, Marjolein Wijnen, Sabina Kef, Mathijs P. J. Vervloed, Bert Steenbergen

**Affiliations:** ^1^ Behavioural Science Institute, Radboud University, Nijmegen, Netherlands; ^2^ De Kleine Prins Onderwijsgroep, Utrecht, Netherlands; ^3^ Faculty of Social and Behavioural Sciences, University of Amsterdam, Amsterdam, Netherlands

**Keywords:** participation, leisure activities, vision impairment, motor impairment, teenager, qualitative study, disability

## Abstract

**Purpose:**

Participation in everyday life activities is important for the development of children and is an important topic in rehabilitation practices. This qualitative study aimed to unveil the perspectives and experiences of teenagers with vision impairments (VIs) or motor impairments (MIs) regarding their participation in leisure activities.

**Materials and methods:**

13 teenagers with VIs and 12 teenagers with MIs (age range: 11–15 years) participated in this study. Data were collected using semi-structured interviews. Verbatim transcripts were analyzed following the steps of the phenomenological approach.

**Results:**

A total of 623 significant statements were identified and assigned to 221 meaning units. Finally, 13 themes emerged. Teenagers with VIs and MIs shared many experiences and perspectives regarding participation in leisure activities.

**Conclusions:**

Teenagers reported that they can sufficiently indicate what they experience as pleasant and sufficient considering their participation, as well as the challenges they encountered, such as their impairment, limited transport possibilities, or concerns from parents. Overall, teenagers with VIs or MIs were generally satisfied with the degree and frequency of their participation in leisure activities and felt sufficiently involved.

## Introduction

Participation in everyday life activities is vital for children's development, psychosocial wellbeing, and competence in physical skills (1, 2). Therefore, it is also an essential goal in rehabilitation services for children with disabilities (3–7). However, children with disabilities face challenges when it comes to participation (2). For instance, children with vision impairments (VIs) have lower participation rates compared to a population-based reference group (8), and children with cerebral palsy tend to participate less in leisure activities than children without motor impairments (MIs) (9). Also, Williams et al. (10) found a negative relation between the extent of impaired functioning and participation among children with intellectual disabilities. This paper seeks to unveil the perspectives and experiences of teenagers with VIs or MIs, without additional disabilities, regarding their participation in leisure activities using a phenomenological approach.

Participation, as defined by the World Health Organization (WHO), involves engaging in life situations that are age-dependent and aligned with an individual's developmental possibilities (11). Some researchers argue that this definition is not comprehensive enough (2, 12, 13). While the term “involvement” suggests a social dimension, participation operationalizations predominantly focus on attendance, that is, simply being present in activities with others. Imms et al. (12) proposed that participation is a combination of both attendance and involvement. While attendance involves the diversity of activities and the act of “being there,” involvement concerns the experience and emotions associated with participation, such as feelings of engagement, social connection, belonging, and motivation. In addition, participation has reciprocal relationships with factors both internal to the individual (ability to express preferences, a sense of self, and competence in performing activities) and external factors (environment and context). In terms of Imms et al. (12), “Context is personal, considered from the perspective of the person participating, and relates to the people, place, activity, objects, and time in which participation is set. […] Environment is external, and refers to the broader, objective social and physical structures in which we live” (p. 20). Future participation is determined by the above-mentioned factors, which at the same time are influenced by past participation experiences.

The list of factors internal to the individual is not exhaustive and varies for each individual (12). Factors such as independence, mobility, self-concept, and peer relations are often considered crucial for participation (2, 14–16). However, people with impairments have to deal with restrictions in leisure activities, as they often depend upon others to participate in activities and need assistance to attend activities due to compromised mobility skills (17, 18). Although help from others and special technologies, such as braille apps for blind children (19) or exoskeletons for children with motor disabilities (20), can alleviate mobility challenges, they do not eliminate the individual's frustration with dependence. Relying on others for mobility only partly solves the participation problem by facilitating presence, but at the same time it can also enhance feelings of dependency, incompetence, and lowered self-esteem (15).

Rehabilitation services play a significant role in encouraging participation of children with disabilities by relying on professional expertise (21) and parental input (2). Still, solely relying on professional and parental perspectives is not enough because they may not necessarily align with the perspectives of the children themselves (21, 22). Children are capable of being involved in their own rehabilitation process (23), and their first-hand experiences can help them gain a deeper understanding of what participation means for them, both practically and emotionally (24, 25). This study addresses precisely this by using a phenomenological approach (26) focusing specifically on children's experiences without the influence of preconceived assumptions from researchers, parents, or rehabilitation professionals (27). Unlike other approaches, the phenomenological approach refrains from theoretical assumptions found in questionnaires (28) and avoids comparisons with control groups or established norms (24). As our research questions concern lived experiences of teenagers regarding participation in leisure activities, the phenomenological approach is pre-eminently appropriate because it can study *what* participants experience and *how* they have experienced it. In this way, authentic meaning can be given to the phenomenon “participation” (26).

There is already an abundance of recent studies describing barriers and facilitators to participation across different groups, including adults with vision impairments (14, 16), children on the autism spectrum (25), and heterogeneous groups with and without developmental disabilities (2, 29). Alongside the phenomenological approach, a unique element of this study is its focus on two specific groups—namely, participation experiences in leisure activities of teenagers with VIs or MIs, without additional disabilities. Different types of disabilities may have varying implications for participation (30). However, such heterogeneity can also exist within a single type of disability (31, 32). Cross-disability research can contribute to earlier and more tailored care for children with disabilities and their families (33), especially because effective interventions require a multidimensional approach. In this study, both groups are limited in their mobility and independence, which may impact their attendance and therefore participation. For children with MIs, these challenges with locomotion has predominant neurologic or motor causes (34), while for children with VIs, the difficulties are likely related to problems with wayfinding, orientation, and obstacle detection (35). The nature of the impairment is not the same in both groups. Yet, children with VIs or MIs sometimes exhibit slow and influential movements and therefore share some experiences with regard to mobility, although the origin of the motor problems might differ (36, 37). However, following the family of participation-related constructs (fPRC) framework by Imms et al. (12), participation is determined not only by physical competences but also by factors such as personal preferences and self-concept. These factors may overlap across disability groups.

Emphasizing the various aspects of participation (12) and investigating the meaning of participation for children with disabilities (3) can assist in prioritizing their needs in early intervention and rehabilitation practices (23, 26). Therefore, this paper aims to uncover teenagers’ perspectives and experiences regarding participation in leisure activities. To gain insight into independence and mobility, the first research question is: (1) How do teenagers with VIs or MIs experience independence and mobility? To further understand participation, the following research questions are posed: (2) What leisure activities do teenagers with VIs or MIs participate in? and (3) How do teenagers with VIs or MIs experience their participation in leisure activities?

## Material and methods

### Participants

Participants, aged 11–15 years and fluent in Dutch or English, were recruited via convenience sampling. Group 1 included teenagers with vision impairments (VIs) from the two Dutch rehabilitation centers for people with VIs and were classified as having severe or moderate low vision based on criteria of the World Health Organization (38). Moderate low vision was considered a visual acuity between 5/100 and 30/100 or a field of vision between 10° and 30°. Teenagers were categorized as having severe low vision when their visual acuity was less than 5/100 or their field of vision was less than 10°. Teenagers who met the inclusion criteria received information about the study, including a flyer and a link to a video clip with audio details. Group 2 consisted of teenagers with a medical diagnosis of a MI and was classified based on the Gross Motor Function Classification System (39). They were recruited from a special education school for children with MI in the Netherlands. Teenagers who met the inclusion criteria received information, including a flyer and a short presentation about the study in class. In case of interest, additional information was offered. Both teenagers and parents were required to provide consent for participation. In both groups, the presence of evident multiple disabilities, which is more than one impairment, each independently leading to disabilities, was an exclusion criterion.

Participants’ demographics are displayed in Table 1. The sample included 25 teenagers, divided into group 1 with VIs (*n* = 13) and group 2 with MIs (*n* = 12). Group 1 consisted of five girls and eight boys, with a mean age of *m* = 14.08 (*sd* = 0.76, range 13–15). Group 2 consisted of seven girls and five boys, with a mean age of *m* = 13.00 (*sd* = 1.15, range 11–14). The specific type of disability was highly varied across both cohorts, such as oculocutaneous albinism and retinopathy of prematurity in group 1 and cerebral palsy and developmental coordination disorder in group 2.

**Table 1:** Demographics of the participants (*N* = 25).

Participant^a^	Age (year; months)	Sex	Degree of vision impairment^b^	Degree of motor impairment^c^
V1	14; 5	Boy	Moderate	—
V2	13; 9	Boy	Moderate	—
V3	13; 2	Girl	Moderate	—
V4	13; 4	Boy	Moderate	—
V5	15; 4	Girl	Severe	—
V6	15; 0	Girl	Moderate	—
V7	14; 11	Girl	Moderate	—
V8	13; 7	Boy	Severe	—
V9	14; 8	Boy	Moderate	—
V10	13; 4	Boy	Moderate	—
V11	13; 5	Girl	Moderate	—
V12	13; 7	Boy	Moderate	—
V13	13; 6	Boy	Moderate	—
M1	12; 11	Girl	—	5
M2	11; 6	Boy	—	1
M3	11; 9	Girl	—	2
M4	11; 8	Boy	—	4
M5	12; 7	Girl	—	1
M6	12; 4	Girl	—	2
M7	12; 3	Boy	—	2
M8	14; 9	Girl	—	1
M9	14; 9	Boy	—	2
M10	13; 4	Girl	—	2
M11	13; 1	Boy	—	2
M12	14; 3	Girl	—	5

^a^ V refers to group 1 (vision impairment); M refers to group 2 (motor impairment).

^b^ Moderate = visual acuity between 5/100 and 30/100 or a field of vision between 10° and 30°. Severe = visual acuity is less than 5/100 or a field of vision less than 10°.

^c^ For consistency, the degree of motor impairment is based on the Gross Motor Function Classification System (39), varying from limited coordination (1) to manual wheelchair in all settings (5).

### Procedure

Participants were interviewed individually by the first author, second author, or student researchers in face-to-face or online video meetings^1^. All participants provided consent for audio recordings of the interviews. Parents provided demographic information about the participants. The interviews started by asking what the participant did last weekend and on a “typical” day in a week. Follow-up questions were asked to gain a deeper understanding of the children's experiences. Examples of questions are as follows: “What do you like about [the activity]?” and “What does being independent mean to you?.” As often as possible, the participants were asked to give examples of what they told to concretize the participants’ answers without prompting them for specific answers. This was especially important to enhance the validity of the participants’ responses (40). Interviews lasted from 13 to 45 min (*m* = 21 min). In total, 25 interviews were conducted. The study was performed in accordance with the ethical standards outlined in the 1964 Declaration of Helsinki and its later amendments or comparable ethical standards. Approval was granted by the Medical Ethical Committee of Eastern Netherlands, Nijmegen, the Netherlands (No. NL74630.091.2 0) for group 1 and the Ethics Committee Social Sciences Radboud University, Nijmegen, the Netherlands (No. ECSW-2021-140) for group 2.

### Data analysis

Interviews were transcribed verbatim, pseudonymized, and then analyzed using the steps of the phenomenological approach in ATLAS.ti (version 23). This method facilitates an authentic understanding and interpretation of the phenomenon of “participation” as experienced by the target group itself (26) and ensures a systematic and in-depth analysis of the data (41). The first step in the phenomenological approach included “bracketing,” meaning excluding the researcher's own feelings and perceptions, to enhance data validity (26)*.* In addition, all authors' backgrounds and contributions were stated in reports (42). The first author was trained as an elementary school teacher and educationalist, with practical experience in teaching children from both regular primary and secondary schools. The second author was also trained as an elementary school teacher, specializing in pedagogical sciences, and currently works at a school for children with motor impairments. The third author holds a PhD in pedagogical sciences. Her research addresses the social-emotional development of adolescents and young adults with vision impairments. The fourth author also holds a PhD in pedagogical sciences. His research addresses the development of young children with vision impairments or multiple disabilities. The fifth author, trained in human movement sciences, holds a PhD in social sciences. One of his research projects focuses on sports and movement of children with motor impairments. In step 2, the first and second authors identified important statements from all participants pertaining to research questions, resulting in 623 significant statements. This was done in a research question-driven way, meaning that we specifically looked for statements related to the research questions. In step 3, the first and second authors assigned general meanings to the significant statements, forming “meaning units” that remained faithful to the original meaning conveyed by the participant. Significant statements with the same tenor were assigned to one meaning unit. The first and second authors created meaning units separately, yielding 221 meaning units after reaching a consensus through discussion. In the fourth step, related meaning units were integrated into themes to develop an in-depth description of the phenomenon. Again, the first and second authors performed this step individually, followed by discussion and consensus. Initially, 17 themes were formed. Subsequently, the third and fourth authors participated in steps 3 and 4, checking the meaning units and verifying the corresponding themes. Based on their judgment, some meaning units were rearranged within the themes, resulting in the final 13 themes. The full methodology is available at the Open Science Framework (https://osf.io/rmuwz/?view_only=ead81624f34045779ba12837e1670ba9).

## Results

**Figure 1:**
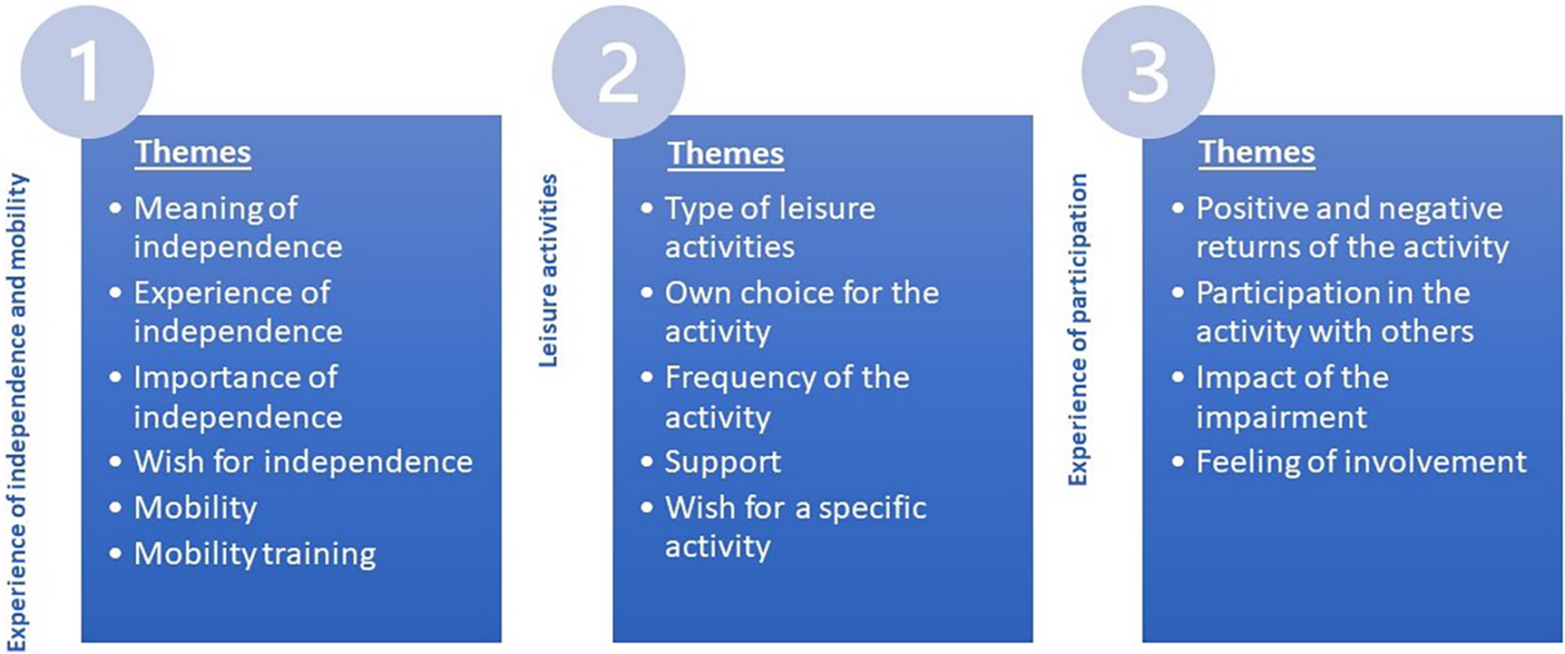
Themes related to the research questions.

In total, 13 themes emerged from the interview, as displayed in Figure 1. To illustrate the process of the phenomenological approach of forming meaning units from statements and subsequently categorizing meaning units in themes, Table 2 presents examples of significant statements and their meaning units that form a theme. The answers of the teenagers to the question regarding types of leisure activities were not used as significant statements for forming meaning units but clustered in Table 3 to give an overview of the leisure activities.

**Table 2:** Examples of significant statements and their formulated meaning units that together form a theme^a^.

Significant statement	Meaning unit	Theme
“*Is it also important for you to be independent?* I don't know. Maybe yes, maybe no.”	Not sure about the wish to be more independent	Importance of independence
“Because I'm usually alone and then, uhm, I have to, so if someone arrives or something, I have to be able to stand up for myself and things like that.”	Because you are often alone, it is important to be able to stand up for yourself	
“Because, for the future it [independence] is very important.”	Independence is becoming more important with age	
“Uhm, yes eventually I also have to be able to do it independently. So then eventually I have to learn it a little bit.”	Independence is becoming more important with age	
“But I do think it's important to, if you learn that too, because later on I also have to do things independently and then it's already useful to learn it a bit myself.”	Independence is becoming more important with age	
“It is important because I am also getting older. And then I have to be able to do things myself and not have my mother cutting my nails when I'm thirty, I have to learn that myself too.”	Independence is becoming more important with age	
“…Pretty important, because you are in grade 6 and then you have to be pretty independent, in my opinion.”	Independence is becoming more important with age	
“*Does that come with age do you think?* Yes. I do think that comes roughly with age. Yes. W*hen you are a bit older, that you become a bit more independent*. Yes.”	Independence is becoming more important with age	
“*How important is it for you to do things outside independently?* Important”	Independence is important	
“Just out alone? *Yes, how important do you think that is?* Somewhat important”	Independence is important	
“I think that's [independence] pretty important.”	Independence is important	
“*How important is it for you to be independent? To do things outside the home?* Not really very important so far. *Yes, okay, and why is it not very important so far?* Because I'm not out of the house very often	Independence is less important if you do not come outside that often	
“Uhm, yes, I don't like help. Preferably, yes preferably I just want to do everything myself. And arrange things myself, so yes, and I get bored really quickly. I'm really someone who, preferably I'd be working until 11 o'clock right now, so to speak. I just like doing things. Not to be bored.”	Independence is important to do things by yourself	
“Ehm, yes because, it is easier if you can do everything yourself rather than always needing help.”	Independence is important to do things by yourself	
“It's also important to do things yourself, so yes, I do think it's important.”	Independence is important to do things by yourself	
“Um yes, that's um, I'm glad for example that I don't have much trouble with my sight that I can do all that independently. But for the rest yes, I don't really think about it either. *No, and then why is that so important to you?* Um, yes because, it is easier if you can do everything yourself than always needing help.”	Independence is important to do things by yourself	
“Pretty important, because I don't always find it necessary for my parents to know everywhere I want and would go.”	Independence is important because parents do not need to know everything	
“It's important though. Because you can't ask someone all the time of you, do this, do that.”	Independence is important because people cannot always help you	
“Pretty important. Because it's not always possible to bring someone with you, you have to be able to do it yourself once.”	Independence is important because people cannot always help you	
“Quite important because people can't always pay attention to you”	Independence is important because people cannot always help you	
“When I go to college, I hope I'll just go home by train, But I already know how to travel by train, so That's not so exciting anymore either.”	During student life you need to travel by train, but I already know	

^a^ Meaning units and themes were based on significant statements of both teenagers with VIs and teenagers with MIs.

**Table 3:** Participation in leisure activities.

	Teenagers with vision impairments	Teenagers with motor impairments
Home-based		
Various individual activities	Drawing, coloring, puzzles (1)	Drawing (2)^a^
	Knitting (1)	Playing or walking with dogs (2)
		Going to animals around the house (1)
		Having a walk (1)
		Taking a bath (1)
		Laze around (1)
Schoolwork	Making homework (10)	Making homework (2)
Digital entertainment	Watching TV (2)	Watching TV (3)
	Watching YouTube (1)	Watching YouTube (1)
	Using the smartphone (1)	Using the smartphone (2)
	Watching a movie (1)	Using the tablet (1)
Gaming	Gaming (with peers) (5)	Gaming (with peers) (1)
	Gaming (company not mentioned) (2)	Gaming (company not mentioned) (1)
		Gaming (sometimes alone, sometimes with peers) (1)
		Gaming (alone) (2)
Home-based activity with the family	Playing games with siblings (1)	Playing games with the parents (4)
	Watching a movie with the household family (1)	Drinking tea with mum (1)
	Helping dad with the pavement around the house (1)	
Outdoor		
Job	Working in the garden (summer) and warehouse (winter) (1)	Doing jobs/chores for money (1)
	Volunteering (1)	Delivering newspapers (1)
	Working at McDonalds; assisting with gymnastics (1)	Photographing (1)
	Assisting with the open day at school (1)	
Musical activity	Piano lessons; singing lessons (1)	Keyboard lessons (1)
Sport—team	Soccer (3)	Soccer (1)
	Korfball (1)	Dancing class (2)
Sport—individual	Swimming (1)	Swimming (2)
	Fitness (1)	Fitness (1)
	Horse riding (2) Judo (2) Karate (1)	Horse riding (2)
Outdoor activity with the family	Going to a brush court (1)	Celebrating the birthday of the mother in a restaurant (1)
	Going to the beach (1)	Went to a market (1)
	Going to the church (2)	Going to a puppy course (1)
	Visiting the grandparents (3)	Watching the swimming class of the siblings while having a drink in the café (1)
	Staying at an aunt (1)	Shopping new clothes (1)
	Going to a cousin's birthday (1)	Watching trains (1)
	Evening walk or forest walk with the household family (1)	Visiting a family member in a clinic and going to the beach (1)
		Going to a grandparents’ birthday (1)
		A walk with the dog and sibling (1)
Cultural activity with peers	Fishing with friends (1)	Girls club (1)
	Scouting (2)	Scouting (1)
	Youth association of the church (1)	
Meeting with friends	Meeting with friends (8)	Meeting with friends (1)
	Shopping with friends (2)	Meeting with a friend outside (1)
		Playing outdoors (1)
		Meeting with one friend (1)
No activity mentioned	Doing nothing because of the cancelation of soccer due to COVID-19 (1)	Doing nothing (1)

^a^ The number of participants who mentioned the specific activity is displayed between brackets ().

### Research question 1: independence and mobility

The first research question explored how teenagers with VIs or MIs perceived independence and mobility. Based on the interviews, we identified six different themes that answered the first research question: meaning of independence, experience of independence, importance of independence, wish for independence, mobility, and mobility training (see also Figure 1).

#### Theme 1: meaning of independence

Without exception, every teenager described independence as being able to function without help from others and doing things on their own. For instance,

“Just being able to do things by yourself, and eeh, arranging things by yourself, and doing things by yourself, you know.” (V9).

According to the teenagers, independence was clearly related to autonomy, including

“Well, that you can live your own life well and yes, be able to do everything yourself. That you don't need the help of others, yes.” (V7).

“That for a while, you don't have people telling you what to do.” (M1).

“Uhm freedom, so just doing what you want and can and not being dependent on everything.” (V6).

Independence was also linked to age, specifically in terms of acting age-appropriately and fostering autonomy from parental influence.

“That I can do things for myself and things that I should actually be able to do myself, that someone my age should be able to do.” (M9).

“Because you can't always expect someone else [mum] to bring you or go with you.” (V6).

#### Theme 2: experience of independence

Teenagers mentioned that they felt both independent and dependent during their leisure activities.

“When I go to school, I feel independent. Because I know where I'm going. And sometimes when I go to a place I don't know, I don't feel independent.” (V5).

“Yes, I can just do many things on my own, no need for my parents to help me with that.” “So, you don't need help with anything?” “No, well, not nowhere with anything.” (V9).

The feeling of independence was sometimes hampered by an overprotective social environment, as participants M11 and M12 mentioned. In addition, a substantial number of teenagers received help. The complexity arises as participants indicated mixed feelings about consistently receiving help. While they expressed a desire to avoid constant assistance, they also valued the presence of peers, creating a nuanced perspective. This is where complexities lie because participants mentioned they do not always want help. Yet, they do not want to do everything independently because the presence of peers is also convivial, as V4 described.

“Mum is far too worried. She is worried that I have to go to the toilet, and no one is there.” (M12).

“I quite like it when people are involved but I don't like it when everyone asks with everything if I need help.” (M11).

“Uhm, doing things outside the door that is important to me, I also find that convivial. But being independent, it's also convivial with friends around you.” (V4).

#### Theme 3: importance of independence

Many teenagers expressed that independence is important to them. Teenagers believed that attaining independence is essential for their transition into adulthood, as they recognized the need to rely less on continuous support from their family. For example, they stated

“I think that’s important for me because I have to learn it myself at some point, because there can't always be someone with me.” (M6).

“… But I do think it's important to learn too, because later on I also have to do things independently and then it's already useful to learn a bit now.” (V2).

“Suppose someone arrives or something, I have to be able to just stand up for myself and things like that.” (M10).

The participants’ statements revealed their aversion to the inevitability of dependence on others. While some teenagers believed independence is an innate quality, others opined that it must be taught by the environment.

“I do think someone will have to teach me then.” (M6).

Whereas all teenagers with VIs felt the urge to be independent, this was not the case for all teenagers with MIs. Some MI teenagers did not feel the urge to become more independent in the near future because they did not go outside without adult supervision. For participants M9 and M12, help was always available.

“Because I am never out on my own.” (M9).

“I never leave home without my mum and dad.” (M12)

#### Theme 4: wish for independence

The statements of a small group of teenagers revealed that in addition to the importance of independence, they had an explicit wish to be more independent, for example,

“…Because I would like to go by myself once without people.” (M1).

“Uhm, yes, I don't like help. Preferably, yes preferably I just want to do everything myself. And arrange things myself.” (V6).

#### Theme 5: mobility

For participants, the theme of mobility intersected with the theme of independence, as being independently mobile had a major impact on teenagers’ feelings of independence. Teenagers described mobility as the ability to move from one location to another; for example, the participants expressed it as

“Uhm, that I can get to where I need to go at a fine pace that suits me. That you can go to it yourself.” (V4).

Yes, yes that is nice when you are just well mobile that you just, yes, you say just I am going there and then I don't need anything else at all.” (V1).

The attainment of independent cycling was emphasized as an important goal. In certain instances, teenagers could only achieve this with the assistance of their parents, like V8. Many teenagers with VIs explained that a lack of overview of the traffic resulted in less mobility, like V7 said.

“If my friend lives here in the village I cycle there with one of my parents.” (V8).

“Then, if something happens, then I, then eeh yes, then maybe I don't react to it so well. Or then I don't see it so well. So then, yes, then sometimes things just don't go so well.” (V7).

The dependency on adults for mobility was highlighted in many interviews. This was particularly evident for teenagers with MIs, as their friends often lived further away due to attending schools for special education outside their hometowns. Consequently, these teenagers needed their parents for transportation to engage in leisure time activities.

“But my father or mother will then take me to the riding school or a meet up with friends.” (M11).

Teenagers who moved around independently sometimes used aids that supported independent mobility. For example, teenagers who used wheelchairs benefitted from electric wheel support, which requires less power and therefore could cover greater distances. Teenagers with VIs could make use of a cane. In addition, several teenagers mentioned that they used a navigation app to plan their routes.

#### Theme 6: mobility training

To enhance mobility, teenagers could follow mobility training initiated by rehabilitation services. It was notable that only teenagers with VIs had specific mobility training during leisure time.

“Uh yeah, I had a few months ago I had mobility training here at [name rehabilitation center], which is the rehabilitation center here, that big one. And it was like to, to learn how to use my cane, for visually impaired and blind people to walk to school.” (V5).

### Research question 2: types of leisure activities

The second research question concerned the types of leisure activities attended by teenagers with VIs or MIs. Table 3 provides the leisure activities mentioned by teenagers with VIs and MIs separately. Some participants mentioned more than one activity. Teenagers from both groups reported participating in home-based activities, often involving family members, such as playing board games. Other frequently mentioned home-based activities included those centered around digital entertainment, such as watching television or using smartphones. The most popular activity was gaming, for both groups. In addition to home-based activities, outdoor activities were also listed. Teenagers participated in sports activities, both individual and team sports, scouting, or gatherings with friends. Overall, teenagers with VIs participated slightly more in outdoor activities, such as sports activities, part-time jobs, and gatherings with friends, than their peers with MIs. In addition to the type of activities, we found four themes related to the types of leisure activities (see Figure 1).

#### Theme 7: Own choice for the activity

Teenagers were asked whether they autonomously selected the leisure activity. The responses revealed that many activities, particularly those at sports clubs, were initiated at an early age, with parents taking the lead. As teenagers grew older, they made independent choices to persist in these activities. When teenagers started with activities later in life, such as having a part-time job, they more frequently expressed having chosen the activity themselves. At times, a rehabilitation organization assisted in selecting an activity, like M8 reported.

“Yes, she investigated with me what I could do and what my hobbies were, and they then referred me to that place.” (M8).

The social aspect was one of the reasons given by teenagers for choosing a particular leisure activity, as interacting with peers and the sociability that comes with the activity held importance for them. Teenagers experienced positive feelings during these leisure activities, such as happiness and fun. In addition to the social aspect, teenagers named relaxation and interest in the activity as essential pillars for their participation. V5 described playing music with the piano as relaxing.

“Playing my piano is quite relaxing, for instance when I come home from school.” (V5).

Several teenagers highlighted enjoyment as a significant factor influencing their activity choices, often associating it with elements like competition or skill enhancement. In addition, they considered their impairment when selecting activities, opting for those that could be performed despite the limitations of an impairment, similar to the experience described by M11.

“Somehow, I’ve always had a thing for horse riding, back in the day from childhood. But I only started riding later because my body was a bit worse then anyway.” (M11).

#### Theme 8: frequency of the activity

Participants were asked what they thought about the frequency of the leisure activities. The majority of teenagers believed that their engagement in leisure activities was adequate. Only a few expressed a desire for more frequent participation. Notably, distinctions emerged between teenagers with VI and those with MI. Most teenagers with VI expressed a desire for an increased frequency, citing enjoyment or a preference for leisure over obligations. On the other hand, some teenagers with MI expressed a similar desire for more frequent participation, but the constraints of fatigue resulting from their MI made it unfeasible.

“I would like to do that more often, but I don't think my body can handle it.” (M11).

#### Theme 9: wish for a specific activity

When being asked what they would like to do when anything would be possible, teenagers referred to a broad range of activities, including low-key and more challenging activities.

“I would love to go to one of those big water parks and cuddle puppies all day.” (M3).

“One time shopping with my grandmother and my mother together.” (V3).

“Driving a tractor, I would love that.” (V11).

“And eeh, I would like to go skydiving with my friends, my blind friends let's say. Because they think that's really cool. Because they are very curious what kind of feeling that gives because they have no idea what that is. So that seems like a really cool thing to do.” (V6).

Talking about their desired activities, both teenagers with VIs and MIs did not mention their impairment. Just a minority was dreaming about activities that they thought were not possible because of their impairment.

“Soccer. Or basketball, just sports.” (M4).

“What do you want to do if anything would be possible? Walk.” (M1).

“Just doing sports with other kids together, that I can do that too.” (V7).

### Research question 3: participation experiences in leisure activities

The third research question entailed how teenagers with VIs or MIs experienced their participation in leisure activities. Four themes were distinguished to answer this research question: Positive and negative returns of the activity, participation in the activity with others, impact of the impairment, and feeling of involvement (see also Figure 1).

#### Theme 10: positive and negative returns of the activity

Almost every teenager indicated that the leisure activity was fun. Other positive expressions were calmness and sociability. The presence of peers could enhance the enjoyment of the activity, M8 reported. The presence of peers is not only related to group activities such as a team sport but also when meeting with friends or during gaming. In the latter, teenagers said they interacted with peers online.

“Having fun with friends and people you know.” (M8).

A sense of autonomy was commonly linked to the leisure activities of the participants.

“Being free and just doing your own thing.” (V2).

“That no one tells me what to do and also something I can do all by myself.” (M1).

Leisure activities were primarily considered as fun. Yet a few teenagers mentioned the downsides associated with the activities. These unfavorable aspects were often attributed to the impairment, such as muscle strain or fatigue.

#### Theme 11: participation in the activity with others

Only some teenagers with MIs mentioned that they preferred to perform activities alone. However, the majority of the teenagers favored participating in leisure activities with others since it is fun and sociable to partake in them together.

“I do like it in a group.” (V1).

#### Theme 12: role of the impairment

The role of the impairment in leisure activities recurred in several ways, as already mentioned in theme 10. Particularly, teenagers with MIs mentioned the challenges associated with their impairment, for example, the need for help in daily life and personal care.

“After school I have to be changed on Sundays I am bathed.” (M1).

For teenagers with MIs, fatigue and muscle strain due to overexertion played a part. These teenagers mentioned this in the following statements.

“I would like to do that more often, but I don't think my body can handle it.” (M11).

“I thought that was enough because I was sore for days afterwards from.” (M7).

While most teenagers had predetermined activities during leisure time, some deliberately avoided making plans after school or during weekends. They attributed this decision to fatigue, which they linked to their impairment. Considering school as a draining experience, they felt the necessity to rest, opting to either lie in bed or stay at home, engaging in low-intensity activities, although these activities were not necessarily preferred.

“Well, the weekend is actually to rest, bit of using the tablet lying in my bed, that I don't have to do much more then. […] My hobby is football, but there I can … I actually can't with my muscles. We tried that but that's not an option.” (M4).

Another drawback of the impairment mentioned by only some teenagers with MIs is that they did not participate in leisure activities after school due to a lack of time. This is because they were taken home by a taxi at the end of the school day. As the taxi transported multiple students, going home took much longer than an individual ride home. M5 explained what he does after school.

“Usually not much, because I get home around four, half past five and then there is not actually much to do.” (M5).

Also, teenagers with VIs commented on the setback of their impairment. For example, V8 said he could not always participate in a soccer match.

“But, at the match, there is then, say, a check on what the score is for how much I can, say, play. Okay, because you have impaired sight? Yes. Okay, so it becomes … You don't always get to play? Well on Tuesdays and Thursdays, yes, but not on Saturdays.” (V8).

Nevertheless, it appears that the challenges encountered by teenagers with VIs during leisure activities were not primarily linked to the activities *per se* but rather to the transportation to and from the activities.

“No, it's just, then we do have to take time into account, because then I have to cycle a bit slower in a larger group.. Uhm, yes sometimes it's a pity, but yes, with my vision impairment it's not really possible sometimes either.” (V4).

#### Theme 13: feeling of involvement

Involvement reflects the extent to which teenagers feel themselves involved in the activities they engage in, as well as their connection to their social environment and their satisfaction with their involvement. All participants indicated that they felt involved in their leisure activities, mentioning several reasons for their involvement. An important factor was the presence of peers, as M11 and V9 described. In addition, V3 indicated that he felt more involved because others showed active involvement and fanaticism.

“Me and my little brother we always do it together and we also share the money, so I do feel involved in it.” (M11).

“Yes, just because I like it, I have a nice team.” (V9).

“Everyone is involved always in games and so on, including me.” (V3).

In addition to the presence of peers, multiple factors contributed to the level of involvement. For example, activities that were specifically focused on the teenager, those requiring a high level of concentration, or those fitting well to their abilities despite their impairment resulted in experiencing a high level of involvement. In addition, teenagers who independently chose their activities experienced a high level of autonomy and fun and therefore felt involved.

“There are always different activities there and some things I cannot see like reading a map and then there are always other activities I can do then.” (V4).

“No, not at all [hard to do the activity], because she even got enlarged cards.” (V6).

“Yes, that's really a lot of fun. Yes, that um, it's fun, very often I get to choose what we're going to do myself too.” (V6).

When the teenagers were restricted in their participation in activities due to their impairment, involvement dropped sharply, V9 explained

“Uhm, because at uh, on Saturday, for example, I can't always play in the match itself. They check what the score is to see how much I can play.” (V9).

## Discussion

The current study explored the viewpoints and lived experiences of 25 teenagers with either VIs or MIs concerning their participation in leisure activities, independence, and mobility. Previous research has revealed disparities in perspectives between children and their parents (43) and rehabilitation professionals (44), with children exhibiting a more optimistic outlook than parents and professionals. In addition to insights from professionals, parents (45, 46), and researchers (12, 47) in prior studies, this study focused on teenagers’ perspectives. The study employed a phenomenological approach (26) to explore teenagers’ shared experiences in leisure activities, avoiding preconceived assumptions (27). The cross-disability approach acknowledged the heterogeneity within the groups (32, 48) and provided insight into the extent and impact of living with a disability; most importantly, it highlighted teens’ experiences regarding participation, mobility, and independence. This approach aligns with the rationale of the individual differences approach (49) and enhances rehabilitation practices by directly considering the perspectives of the target population, thus ensuring better alignment with their needs (15). In addition, with this empirical information, conceptual models such as the fPRC framework (12) can be complemented, giving meaning to factors like “preferences” and “the environment.”

The first research question focused on elucidating the teenagers’ definition of independence and mobility. Without exception, independence was defined as the ability to perform tasks without needing immediate assistance from others. It became evident that, for these teenagers, mobility was intricately linked to independence. The expression of personal preferences aligns with one of the intrinsic factors associated with participation in the fPRC framework (12). That independence is a crucial theme for teenagers with disabilities is also evident from the multitude of themes that have emerged, encompassing various aspects of independence: the meaning of independence, the experience of independence, the importance of independence, and the wish for independence.

The second research question explored the types of leisure activities in which teenagers with VIs or MIs engaged in. Various activities were mentioned, with gaming as the most popular one. As Stone et al. (50) concluded for youngsters on the autism spectrum, the popularity of gaming among teenagers may stem from the opportunity to interact with peers conveniently from home, which fosters social participation (51). The convenience for the target group in this article, children with VIs or MIs, might also related to the fact that games can be played from home, overcoming mobility problems.

The findings of the third research question, how do teenagers with VIs or MIs experience their participation in leisure activities, indicate that both groups generally perceive their participation in leisure activities positively, consistent with the review of Powrie et al. (52). Participants emphasized aspects such as having fun and interaction with peers, which align with the intrinsic fPRC factor of sense of self (12) and mirror results from a study involving adults with VIs, who mentioned fun as the most important motive to participate in runs (53). In line with previous research (54, 55), some participants experienced limited participation due to a direct consequence of the impairment, such as functional limitation, pain, or fatigue. In this respect, these limitations negatively affected the fPRC factor of activity competences (12). Teenagers also cited indirect effects of their impairments, such as limited transportation options or parental concerns, as the reasons why they were hindered in leisure time activities (54), related to the fPRC factor of environment (12).

Although this study primarily focused on many similarities, we noticed some differences in the experiences of the two groups. Specifically, whereas all teenagers with VIs expressed a desire for independence, this sentiment was not uniformly shared by teenagers with MIs. Moreover, diverse barriers to participation in leisure activities were identified. Teenagers with MI highlighted obstacles such as pain, fatigue, muscle strain, and time constraints. In contrast, transportation emerged as the primary concern for teenagers with VIs, which aligns with the findings of Wright et al. (44) and Jaarsma et al. (56). Nevertheless, experiences and perspectives were mostly similar instead of different. On the basis of this study, the nature of the disability seems to matter little for participation in leisure activities.

### Implications

The current study shed light on teenagers’ perspectives regarding participation as a pivotal component in rehabilitation (3, 5–7). To engage teenagers with VIs or MIs, emphasis on the social dimension is necessary. The identified values commonly associated with leisure activities, such as “fun” and “sociability,” highlight the importance of tailoring activities that align with these intrinsic motivations. The current study acknowledges the reciprocal relationship between intrinsic factors and participation (12). Investing in activities that match the specific preferences of these teenagers can make them more appealing overall. Participating in “fun” activities can be used to develop skills and competencies. For example, games can improve motor skill development (57).

Teenagers value autonomy and the ability to express preferences, linking the feeling of independence to decision-making. Notably, self-chosen activities were consistently associated with a sense of enjoyment. This observation aligns with insights from self-determination theory (58), describing that human motivation is driven by the fulfillment of psychological needs including autonomy, competence, and relatedness. The interviews showed that excessive assistance can undermine feelings of autonomy. Their understanding of independence involved the ability to perform tasks unaided. The ability to make personal choices and the experience of autonomy emerged as crucial pillars for fostering participation, as noted by Imms et al. (12) and Saebu et al. (59).

Previous research on adolescents who are deaf or hard of hearing suggests that a combination of online and offline interaction can strengthen existing friendships (60). In the current study, a substantial proportion of the participants identified gaming as their favorite leisure activity, valuing (online) interaction with their friends. Incorporating online games into rehabilitation practices can prove to be a valuable complement, particularly when individuals not only engage independently but also connect with friends online during games, in addition to real-life interactions. The utilization of online games can promote interaction with peers (50) and social participation (51).

This study demonstrates knowledge about what teenagers want and consider necessary regarding independence and participation. It is, therefore, highly essential for stakeholders, including educators, rehabilitation professionals, and researchers to engage in ongoing dialog with teenagers, as also emphasized in the review of Paul et al. (15) on inclusive education. The themes identified in this research provide valuable insights for initiating conversations with teenagers. These dialogs may encompass pragmatic considerations, such as transportation logistics and perceptual dimensions of teenagers, clarifying their conceptualizations and visions.

### Limitations

Using convenience samples of children with impairments, as in the present study, carries the risk of sample incomparability. In this study, teenagers with VIs attended regular education alongside peers without impairments, while teenagers with MIs were enrolled in special education, where all students have impairments. This educational setting difference may affect participation levels, as larger and/or regular schools tend to increase participation in activities (61–63). Moreover, positive peer attitudes toward impairments are more common in inclusive education settings (64). Consequently, teenagers with VIs might experience these positive peer attitudes more frequently than teenagers with MIs because they are taught within regular education settings. It is further known from the literature that peer attitudes influence the leisure activities of teenagers with disabilities (65, 66). If teenagers are engaged and encouraged by peers to participate, they might show more and more frequent participation (17, 65). However, current research did not consider the impact of education settings on participation.

The study explicitly involved teenagers with a single disability, either VIs or MIs, deliberately excluding teenagers with both VIs and MIs. This exclusion was intentional, as having multiple disabilities does not simply mean that addressing each disability will resolve all issues (67). In fact, the combination of multiple disabilities often leads to more complex and severe challenges. Children with multiple disabilities generally require more extensive support than those with a single disability, who tend to function more independently (68), and may face fewer limitations in daily activities (69). Thus, the current findings are relevant to children with either VIs or MIs, emphasizing their experiences and potential for participation. The study highlights the value of a cross-disability perspective (70) and promotes the development of more effective, individualized interventions (33). However, the study did not address teenagers with both VIs and MIs. While the current findings of the study provide a foundation for further research, they are not comprehensive enough to fully address the needs of teenagers with multiple disabilities. Given the complexities of multiple disabilities, specific research targeting this population is warranted (67–69) to provide more tailored implications for their care and support.

Furthermore, even with using focused research questions to guide the study and to prevent the focus of the study from becoming too broad (26), this procedure may have the consequence that other information participants shared might have been left out, although it could be important to understand participation in leisure activities (41).

Finally, the phenomenological approach assumes that participants have experienced the phenomenon under investigation (26). While heterogeneity exists among the participant groups, this study and previous research indicated that shared experiences outweighed differences, suggesting common perceptions regardless of impairment cause or severity (48, 71).

## Conclusion

The findings of this research described the meaning of independence, mobility, and participation in leisure activities for teenagers with VIs or MIs without additional disabilities. Teenagers can sufficiently indicate what they experience as pleasant and sufficient considering their participation and the challenges they encounter. For example, teenagers with VIs or MIs were generally satisfied with the degree and frequency of their participation in leisure activities and felt sufficiently involved. However, teenagers with impairments were also good at naming factors that hinder participation, such as their impairment, limited transport possibilities, or concerns from parents. The study showed how teenagers experienced participation, which complements the perspective of parents, professionals, and researchers. With this, this research adds a new perspective to the concept of participation, namely, of teenagers with impairments.

## Data Availability

The datasets presented in this study can be found in online repositories. The names of the repository/repositories and accession number(s) can be found here: https://osf.io/rmuwz/?view_only=ead81624f34045779ba12837e1670ba9.

## Footnotes

^1^Due to COVID-19 restrictions, in some cases, we were restricted to plan online video meetings to perform the interview instead of a face-to-face meeting.

## Appendix A

The first author was trained as an elementary school teacher and educationalist. She has practical experience in teaching children from both regular primary and secondary schools and now works as a PhD student. The second author was also trained as an elementary school teacher, specializing in pedagogical sciences. She works at a school for children with motor impairments. The third author holds a PhD in pedagogical sciences. Her research addresses the social-emotional development of adolescents and young adults with vision impairments. The fourth author also holds a PhD in pedagogical sciences. His research addresses the development of young children with vision impairments or multiple disabilities. The fifth author was trained in human movement sciences and holds a PhD in social sciences. One of his research projects focuses on sports and movement of children with a motor impairment. Contributions of all authors are based on the Contributor Role Taxonomy (CRediT) (42).
